# Low intrinsic capacity is associated with risk of developing mild cognitive impairment in the English Longitudinal Study of Ageing (ELSA)

**DOI:** 10.1007/s11357-025-01960-8

**Published:** 2025-11-09

**Authors:** Rosa Birchenough, Ingrid Buller-Peralta, Alejandra Marroig, Graciela Muniz-Terrera

**Affiliations:** 1 Medicine (MBChB), Edinburgh Medical School, College of Medicine and Veterinary Medicine, University of Edinburgh, Edinburgh, UK; 2 Edinburgh Dementia Prevention, Centre for Clinical Brain Sciences, University of Edinburgh, Edinburgh, UK; 3 Outpatients Department Level 2 Western General Hospital, Crewe Rd S, Edinburgh, EH4 2XU UK; 4 Instituto de Estadística, Universidad de La República, Montevideo, Uruguay; 5 Ohio University Heritage College of Osteopathic Medicine, 191 W Union St, Athens, OH 45701 USA

**Keywords:** Global cognition, Item-response theory, Mini-mental State Examination, Healthy ageing

## Abstract

Intrinsic capacity (IC) is a composite that includes five different domains related to a person’s capacities: sensory, locomotion, vitality, psychological and cognitive. IC represents part of a global effort to promote healthy ageing, one aspect of which is healthy cognitive functioning. This study aimed to elucidate the association of IC and cognitive decline in older adults. Using data from the English Longitudinal Study of Ageing (ELSA, wave 6), IC composite was derived through z-score and item-response theory (IRT) methods in a sample of cognitively normal older adults (*n* = 731, aged 70.1 ± 6.77 years). Global cognition (GC) was derived by categorizing Mini-mental State Examination scores obtained in the same population 4–5 years later (ELSA-HCAP, 2018). After algorithm selection, GC categories were regressed on IC scores, demographic and lifestyle covariates. The IRT algorithm showed better model fit and strong associations with age, sex and education. Lower IC was associated with higher likelihood of developing mild cognitive impairment (MCI) 4 to 5 years after IC assessment. Increased age and lower education were also strong predictors of GC, whereas no significant effects were found for sex, physical activity, or number of comorbidities in the fully adjusted model. Although bivariate regressions showed that reduced moderate or vigorous physical activity and increased number of comorbidities are associated with higher odds of developing later MCI, the lack of associations between these covariates and GC in the fully adjusted model suggests a possible mediation effect on IC. No study had previously explored the association of IC on later cognitive impairment. Results agree with previous evidence associating low IC to females, older ages and lower education. Further research to reveal possible underlying mechanisms explaining this association is needed to improve our understanding and relevance of IC for healthy ageing.

## Introduction

The World Health Organization (WHO) defined intrinsic capacity (IC) as “a composite of all the physical and mental capacities that an individual can draw on” [1]. This definition focuses on the resources, rather than on deficits of a person [2], encompassing five interrelated domains to create an overall composite that will reflect essential functional abilities for healthy ageing. In line with the WHO proposal, and a more holistic view of health that goes beyond the absence of physical illness, IC subdomains have been defined as follows: locomotion, psychological wellbeing, cognition, sensory and vitality [3, 4].

Although there is no consensus on a particular methodology to derive an IC score [5], common approaches include confirmatory factor analysis (CFA), the item response theory method (IRT) or summation of z-scores calculated from the indicators measuring the IC domains [4, 6–9]. Regardless the methodology, results shown by previous evidence are consistent, with high IC scores strongly associated with reduced rates of mortality and hospital admissions, as well as preserved activities of daily living and instrumental activities of daily living [8, 10, 11]. However, no research to date has assessed for associations between IC and the risk of developing cognitive impairment or dementia.

Our study analysed data from the English Longitudinal Study of Ageing (ELSA), a population-based longitudinal study of older adults in the UK, to evaluate the association between IC assessed by wave 6 (2012–2013) and global cognitive function measured 4 to 5 years later (2018). We hypothesise that IC will be negatively associated with global cognition in older adults later in life.

## Methods

### Study design

This study used data from wave 6 of ELSA (2012–2013) to derive an IC score, and Mini-mental State Examination (MMSE) scores [12], provided by the ELSA-HCAP sub study (2018) to investigate whether global cognitive function can be predicted by IC measured between 5 and 6 years previously using linear regression models.

### Datasets

#### ELSA study

ELSA is a population-based longitudinal study of individuals aged 50 and over living in the UK. The original dataset contained 11,578 households and 18,813 individuals, and the ELSA dataset was refreshed at waves 3, 4, 6, 7 and 9 using respondents from the HSE [13].

#### ELSA-HCAP sub-study

The ELSA Harmonised Cognitive Assessment Protocol (ELSA-HCAP) is a sub-study of ELSA, implemented between waves 8 and 9 [14]. This sub-study aimed to measure cognitive impairment and dementia prevalence of the older ELSA participants.

### Participants

**Fig. 1:**
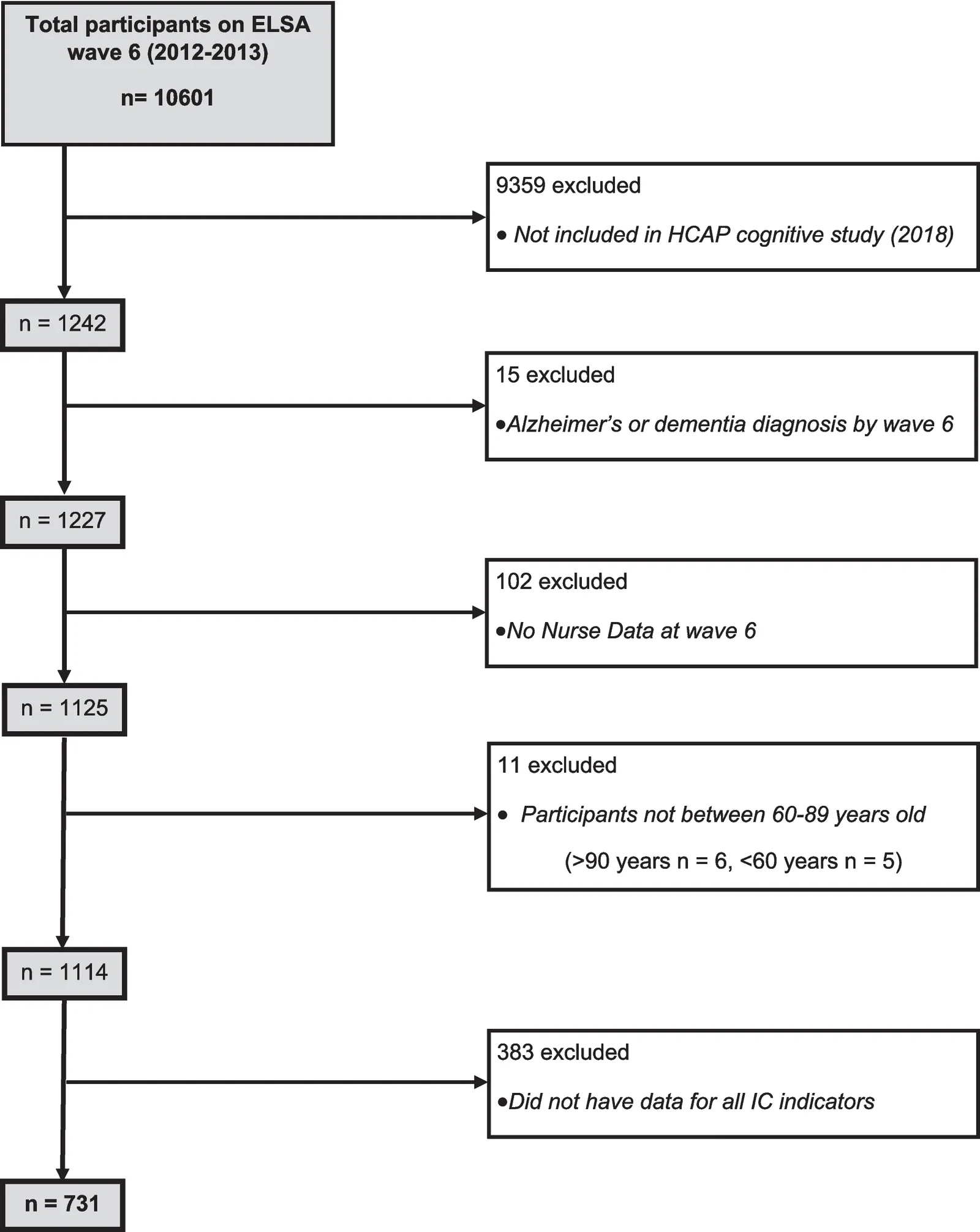
Sample selection criteria

In the current study, participants were selected from ELSA wave 6 (2012–2013). Participants were excluded if they had not taken part in the ELSA-HCAP and had been diagnosed with dementia or Alzheimer’s disease by wave 6. Participants without nurse data available were also excluded. Only participants aged 60–89 years were included to avoid missing data for gait speed, which was not available for ≥ 90 years (*n* = 6). Note also that according to ELSA codification, all participants aged ≥ 90 years are collapsed into one nominal category, limiting the possibility to draw accurate conclusions when age is considered a continuous variable. Participants aged < 60 (*n* = 5) were excluded according to the definition of older adults by the WHO (aged 60 or over). Lastly, participants who did not have data for all the IC variables available were excluded from the study. The final study involved 731 participants (see Fig. 1 for further details).

### Ethical statement

All participants gave written consent prior to the ELSA study. ELSA wave 6 received ethical approval from the NRES Committee South Central—Berkshire on 28th November 2012 (11/SC/0374). For the ELSA-HCAP sub-study, ethical approval was given from the South Central-Berkshire National Health Services (NHS) Research Ethics Committee and was conducted in accordance with the ethical standards of the Declaration of Helsinki. Informed verbal consent was obtained from all participants or their guardians [14].

### Intrinsic capacity indicators and domains

To create a comprehensive IC score, 12 of the variables measured in ELSA were selected to represent the five domains: cognition, locomotion, psychological wellbeing, sensory and vitality. Domains, indicators and cut-off points used in the item response theory (IRT) method are summarised in Table 1.

**Table 1:** Intrinsic capacity domains, indicators and summary scores

Domain	Indicator	Cutt-off scores for impairment [ref]	Raw scores (mean ± SD)			Dichotomous scores (% impaired)		
			Total	Male	Female	Total	Male	Female
Cognitive	**Temporal orientation** (day of the week, day, month, and year. 1 point each, max = 4)	< 4 [9, 21]	3.81 ± 0.43	3.81 ± 0.44	3.81 ± 0.42	17.4	17.5	17.3
	**Delayed recall** (words recalled, max = 10)	Bottom tertile of scores [9]	4.68 ± 1.89	4.50 ± 1.81	4.84 ± 1.95	24.2	25.4	23.2
Sensory	^**a**^** Eyesight** (self-reported, 1 = excellent, 5 = poor)	> 3 [9]	2.44 ± 0.87	2.33 ± 0.86	2.54 ± 0.86	9.03	6.12	11.6
	^**a**^** Hearing** (self-reported, 1 = excellent, 5 = poor)	> 3 [9]	2.71 ± 1.06	2.83 ± 1.08	2.60 ± 1.03	22.3	27.1	18
Vitality	**Grip strength** (Kg)	< 30 kg for males, < 20 kg for females [9, 22]	30.55 ± 7.44	36.12 ± 6.50	35.63 ± 3.91	13.1	18.1	8.76
	**Forced expiratory volume—FEV** (Lts/Sec)	Bottom quartile [7]	2.34 ± 0.69	2.71 ± 0.72	2.01 ± 0.47	25	12.2	36.3
Locomotion	**Balance test** (tandem, semi-tandem and full tandem, max = 4)	< 4 [9]	3.80 ± 0.53	3.84 ± 0.47	3.77 ± 0.58	15.6	12.8	18
	^**a**^** Chair stand** (Sec to complete 5 rises)	> 16.7 s [9]	11.65 ± 3.97	11.43 ± 3.81	11.83 ± 4.11	9.58	8.45	10.6
	^**a**^** Gait speed** (Mts/Sec)	< 0.8 [9, 22]	1.15 ± 0.31	1.16 ± 0.31	1.13 ± 0.31	12.7	11.4	13.9
Psychological	^**a**^** Sleep (**Jenkins Sleep Problems Scale, 1 = not during the last month, 4 = 3 or more times a week, max = 12)	> 6 [7, 23]	6.64 ± 2.51	5.91 ± 2.28	7.28 ± 2.52	46.9	32.7	59.5
	^**a**^** Depression** (CES-D, max = 8)	> 3 [9, 25]	1.05 ± 1.72	0.74 ± 1.39	1.32 ± 1.92	9.3	5.25	12.9
	^**a**^** Satisfaction with Life Scale-SWLS** (scale reversed 1 = strongly agree, 7 = strongly disagree; max = 35)	> 20 [9]	13.68 ± 5.76	13.24 ± 5.53	14.08 ± 5.93	13.8	12.8	14.7

^a^Higher scores represent worst performance

#### Cognition domain

Temporal orientation and delayed recall variables were used. Temporal orientation involved asking the participant to orientate themselves regarding year, month, day and time [8]. A score of less than 4 (maximum score) was considered impaired [8, 15]. Delayed recall was assessed using lists of nouns given aurally to the participant who must recall as many as possible from memory after a short delay [6]. The bottom tertile of scores was considered impaired [8].

#### Locomotion domain

Gait speed, the Balance test (part of the Short Physical Performance Battery) and the Chair-Stand test were used. Gait speed was measured as the time taken in seconds to walk a certain distance (2.4 m) at the participant’s regular pace [6]. A score of less than 0.8 m/s was recorded as impaired [8, 16]. Static balance was measured using balance tests from the Short Physical Performance Battery. These included a side-by-side stand, a semi-tandem stand and a full-tandem stand [6]. A cut-off score of less than 4 was classed as impaired [8]. The Chair-Stand test was measured as the time taken to repetitively rise from and sit down in a chair 5 times without the participant using their hands. The time taken to reach full standing position was recorded in seconds [6]. Five rises in more than 16.7 s were used as a cut-off score and considered impaired [8].

#### Psychological domain

Sleep, depression and satisfaction with Life were included in the psychological domain. The Jenkins Sleep Problem Scale [17] was used to measure sleep and involved questions such as “how many times during the last month had the participant experienced trouble falling asleep”. This was reported in categorized answers [17], and a score of over 6 was considered impaired. Depression was assessed using a self-reported depression scale with 8 items [18] where a score of over 3 was considered impaired [8, 19]. Satisfaction with Life was measured using the Satisfaction with Life Scale [20]. This involved a self-reported questionnaire asking questions such as “in most ways my life is close to my ideal”. Answers are categorized into 7 categories from strongly disagree to strongly agree, and overall scores range from extremely satisfied to extremely dissatisfied [20]. Following a previous approach, the scale was reversed for higher scores to represent lower satisfaction levels, setting a cut-off score of over 20 points for impairments [8].

#### Sensory domain

Sensory was assessed through Eyesight and Hearing. Eyesight was measured via self-reported questions such as “how good is your eyesight for seeing things at a distance?”, with categorized responses ranging from 1 (excellent) to 5 (poor) [6]. A score of 4 or 5 was considered impaired [8]. Hearing was also assessed via self-reported questions, where participants were asked to rate their hearing in categorized responses [6]. As before, a score of 4 or 5 was considered impaired [8].

#### Vitality domain

Vitality was assessed through Forced Expiratory volume (FEV) and Hand Grip Strength. Forced expiratory volume was the maximum volume of air expired by the participant in 1 s using a spirometer [6]. The bottom quartile of results was considered impaired. Hand grip strength was measured using a handheld dynamometer [6]. Each hand (dominant and non-dominant) was trialled 3 times and averaged. The value from the dominant hand was used [6]. A score of less than 30 kg in men or less than 20 kg in women was considered as impaired [8, 16].

### Intrinsic capacity scores derivation

#### Z-score algorithm

The continuous nature of the variables available for each of the domains facilitated the derivation of an IC using z-scores, where a higher score indicated higher intrinsic capacity. Measurements for eyesight, hearing, chair stand, gait speed, sleep, depression and satisfaction with life were reverse coded. z-scores of each raw variable were calculated and z-scores belonging to each domain were then summed and averaged. This value was taken to be the overall score of that domain, and all domain scores were finally summed to create an overall IC z-score, giving equal weighting to all domains.

#### Item response theory (IRT) algorithm

A second algorithm to calculate the IC was implemented using cut-off scores for each of the 12 variables to create dichotomous indicators. Impairment for each variable was coded as 0, whilst capacity was coded as 1 (see details in Table 1**)**. Using these dichotomous variables, an Item Response Theory (IRT) model was applied to calculate a value for the underlying trait for each participant, labelled as intrinsic capacity [21]. Specifically, a 2-parameter logistic model was applied to capture both the difficulty and discriminations of each item.

### Global cognition (GC)—Mini-mental State Examination (MMSE)

The MMSE is a widely used screening test to assess global cognitive function [22]. It consists of 30 questions, with total scores comprising integer values between 0 and 30, where higher values indicate better cognition [12]. From the MMSE total score, it is also possible to categorize the global cognitive status of individuals ranging from normal to severe impairment [23], with high sensitivity for moderate to more severe cognitive decline [24]. Given the lack of normality and ceiling effects of MMSE scores, associations with IC were assessed against these previously defined global cognitive status categories: *Normal* = MMSE scores between 30 and 24, *Mild impairment* = MMSE scores between 23 and 19, *Moderate impairment* = MMSE scores between 18 and 10, *Severe impairment* = MMSE scores between 9 and 0 [23]. Moreover, given MMSE is generally used as a cognitive screening to differentiate normal vs impaired global cognition, an approach using established categories can provide more precise information regarding relevant cognitive changes that the continuous scale cannot detect.

### Covariates

Sociodemographic and lifestyle covariates included age (measured at the time of the ELSA-HCAP assessment), sex, education, smoking and frequency of engagement in physical activity. Diagnosed conditions of lung disease, cancer, Parkinson’s disease, psychiatric conditions, heart attack (including myocardial infarction and coronary thrombosis), brain stroke, hypertension, diabetes and high cholesterol levels were dichotomously scored as present/diagnosed = 1, or absent = 0, to obtain a summed score of comorbidities frequently associated with age-related decline. Given the wealth of evidence supporting a strong correlation between socioeconomic status (SES) and educational level in the English population that has been maintained since the beginning of the last century to date [25], these covariates were not included to avoid multicollinearity issues. Moreover, this trend is confirmed by the 2024 Organisation for Economic Co-operation and Development (OECD) report, showing higher educational levels positively correlated with SES and income [26].

### Statistical analysis

The two versions of IC scores were derived through the R Statistical Software (v4.3.2) [27]. For the 2-parameter IRT model IC scores, the *ltm* package was used [28]. Statistical analyses were conducted using IBM SPSS Statistics v.27.0 [29].

To select the best IC scoring algorithm, each version of IC scores was regressed separately on age, sex and education, given previous evidence reporting strong associations to these covariates [6, 30]. For this analysis, age at the time of IC indicators assessments was considered. Given that the MMSE test contains similar questions to those included within the IC cognition domain, the MMSE score was also included as an additional covariate to evaluate potential collinearities with the IC algorithms. Model assumptions were examined following recommended practice [31, 32].

Overall model fit and model selection were determined by the highest adjusted *R*^2^, lowest Bayesian Information Criterion difference (ΔBIC) > −10 [33], the highest log-likelihood value and greater effect size (Cohen’s *f*^2^) [34].

After algorithm selection, the association between IC and GC categories was assessed through a Multinomial Logistic Regression (MLR) model, with the *Normal* category set as reference. Since only two participants were categorized with severe impairment, this category was collapsed into *moderate to severe* impairment. Age at time of MMSE assessment, sex, education, smoking status, frequencies of mild, moderate and vigorous physical activities and the number of comorbidities were included as covariates.

To assess the individual effects of IC and all the covariates included in the fully adjusted model, bivariate logistic regressions were conducted between GC categories. To discard potential lack of statistical power leading to false negative results in effect of IC on the less frequent GC category (*moderate to severe*), an additional fully adjusted MLR analysis was performed collapsing *mild* and *moderate to severe* GC categories into *impaired GC*.

Multiple comparison analysis of IC between cognition categories was evaluated by the Kruskal–Wallis test for independent groups, followed by the Bonferroni corrected Dunn’s test for multiple comparisons (*α* = 0.05).

## Results

### Participants

The analytical sample included 731 participants (53.1% females), selected from the ELSA wave 6 and HCAP sub-study as described in Fig. 1. The mean age at wave 6 was 70.09 (SD = 6.70) years old (females, 69.63; SD = 6.64), and the average age at the HCAP sub-study was 74.09 (SD = 6.71) years old (females, 73.60; SD = 6.69). Around 98% of participants were non-smokers, and 47.6% had an educational level corresponding to upper secondary or vocational training and had an average of 1 health condition (0.95, SD = 0.99), though they ranged from none to five conditions. During collection of HCAP data, 23 participants had a clinical Alzheimer’s or dementia diagnosis, and 110 participants had some degree of cognitive impairment. 85% had normal cognition according to the MMSE categories, with 12.7%, 2.05% and 0.27% showing mild, moderate and severe impairment, respectively. Demographic descriptions for IC indicators and domains are summarised in Table 1. For detailed demographic characteristics, see Table 2.

**Table 2:** Demographic characteristics

		Total	Male	Female
Sex, *n* (%)		731	343 (46.9)	388 (53.1)
Age at wave 6 (2012–2013), mean (SD) [Range]		70.09 (6.70) [60–89]	70.62 (6.73) (60–89]	69.63 (6.64) [60–88]
Age at HCAP (2018), mean (SD) [Range]		74.09 (6.710) [62–90]	74.65 (6.71) [62–90]	73.60 (6.69) [63–90]
Education, *n* (%)	Less than lower secondary	210 (28.7)	75 (21.9)	135 (34.8)
	Upper Secondary and vocational training	348 (47.6)	184 (53.6)	164 (42.3)
	Tertiary education	107 (14.6)	68 (19.8)	39 (10.1)
	No formal education	66 (9.02)	16 (4.66)	50 (12.9)
Smoking history, *n* (%)	Non-smokers	713 (97.5)	336 (98.0)	377 (97.2)
	Smokers (past or current)	18 (2.46)	7 (2.04)	11 (2.84)
Vigorous physical activity (Frequency), *n* (%)	Hardly ever or never	408 (55.8)	167 (48.7)	241 (62.1)
	1–3 times a month	172 (23.5)	94 (27.4)	78 (20.1)
	Once or more a week	151 (20.7)	82 (23.9)	69 (17.8)
Moderate physical activity (Frequency), *n* (%)	Hardly ever or never	74 (10.1)	31 (9.04)	43 (11.1)
	1–3 times a month	156 (21.3)	62 (18.1)	94 (24.2)
	Once or more a week	501 (68.5)	250 (72.9)	251 (64.7)
Mild physical activity (Frequency), *n* (%)	Hardly ever or never	38 (5.20)	27 (7.87)	11 (2.84)
	1–3 times a month	78 (10.7)	60 (17.5)	18 (4.64)
	Once or more a week	615 (84.1)	256 (74.6)	359 (92.5)
Comorbidities at wave 6, mean (SD)		0.95 (0.99)	1.01 (1.06)	0.9 (0.95)
Lung disease (chronic lung disease such as bronchitis or emphysema), *n* (%)		24 (3.28)	15 (4.37)	9 (2.32)
Cancer (any kind including malignant), *n* (%)		21 (3.83)	14 (4.08)	7 (1.80)
Parkinson’s disease, *n* (%)		3 (0.410)	3 (0.875)	0 (0.00)
Psychiatric conditions		67 (9.17)	21 (6.12)	46 (11.9)
Heart attack (including myocardial infarction or coronary thrombosis), *n* (%)		40 (5.47)	30 (8.75)	10 (2.58)
Brain stroke (cerebral vascular disease), *n* (%)		25 (3.42)	13 (3.79)	12 (3.09)
Hypertension, *n* (%)		258 (35.3)	124 (36.2)	134 (34.5)
Diabetes, *n* (%)		56 (7.66)	30 (8.75)	26 (6.70)
High cholesterol, *n* (%)		204 (27.9)	98 (28.6)	106 (27.3)
Alzheimer’s or dementia diagnosis, *n* (%)		23 (3.15)	10 (2.92)	13 (3.35)
MMSE score, mean (SD)		26.6 (3.41)	26.83 (3.23)	26.4 (3.55)
Global cognition category	Normal, *n* (%)	621 (85.0)	297 (86.6)	324 (83.5)
	Mild cognitive Impairment, *n* (%)	93 (12.7)	40 (11.7)	53 (13.7)
	Moderate Impairment, *n* (%)	15 (2.05)	5 (1.46)	10 (2.58)
	Severe Impairment, *n* (%)	2 (0.273)	1 (0.29)	1 (0.26)

### IC algorithms comparison and selection

IC scores derived through the IRT, and the z-score methods were regressed separately for model fit comparison and algorithm selection. Multiple regressions showed satisfactory fitting for both scoring algorithms. However, the IRT IC score displayed a higher log-likelihood (ΔLL = 626.87) and a substantial decrease of the Bayesian Information Criterion (ΔBIC = −1253.74), compared to the z-score method. Both the z-score and IRT-derived scores showed significant negative associations with age (β = −0.232, *p* < 0.001; β = −0.306, *p* < 0.001), female sex (*β* = −0.241, *p* < 0.001; *β* = −0.152, *p* < 0.001), and MMSE scores (*β* = 0.15, *p* < 0.001; *β* = 0.177, *p* < 0.001). However, a significant positive association between IC and level of education was only evidenced in the IRT algorithm (*β* = 0.095, *p* = 0.007; versus z-score *β* = 0.063, *p* = 0.083). Although the adjusted *R*^2^ of the IRT-derived score was slightly higher than corresponding adjusted *R*^2^ of the z-score (Δ*R*^2^ = 0.04) and both size effects were moderate (Cohen’s *f*^2^ = 0.21 and 0.27 for z-score and IRT, respectively), the IC score derived through the IRT model was selected for proving better fit criteria. No collinearities were found between variables. See Table 3 for full model fit and effect size comparisons between IC scorings.

**Table 3:** Model fit and effect size comparisons between IC scoring algorithms

Goodness of fit	z-score	IRT-score
Log Likelihood^a^	**−**1309.74	**−682.87**
BIC^b^	2659.05	**1405.31**
Model summary		
*R*^2^	0.173	0.213
Adj *R*^2^	0.169	**0.209**
SE	1.457	0.618
Model Fit F(4,726)	38.02	49.14
*p*	< 0.001	** < 0.001**
Effect size^c^	0.209	**0.271**
Coefficients		
*Age*	*β* = −0.232, *p* < 0.001	*β* = −0.306, *p* < 0.001
B [95% CI]	−0.055 [−0.072 −0.038]	−0.032 [−0.039 −0.025]
VIF^d^	1.135	1.135
*Sex*	*β* = −0.241, *p* < 0.001	*β* = −0.152, *p* < 0.001
B [95% CI]	−0.771 [−0.991 −0.552]	−0.212 [−0.305 −0.119]
VIF^d^	1.075	1.075
*Education*	*β* = 0.063, *p* = 0.083	*β* = 0.095, *p* = 0.007
B [95% CI]	0.121 [−0.016 0.257]	0.080 [0.022 0.137]
VIF	1.152	1.152
*MMSE*	*β* = 0.15, *p* < 0.001	*β* = 0.177, *p* < 0.001
B [95% CI]	0.07 [0.037 0.104]	0.036 [0.022 0.05]
VIF^d^	1.169	1.169

Bold numbers denote significant values with *p* < 0.05

*β* standardized coefficients, *B* unstandardized coefficients and their respective CIs

^a^Greater values show better goodness of fit

^b^Bayesian Information Criterion. Smaller values show better goodness of fit

^c^Cohen’s *f*^2^, where values ≥ 0.15 show moderate effects and ≥ 0.35 show large effects

^d^Variance inflation factor. Values < 2 indicate no collinearity

### Associations between global cognition and IC

Associations between global cognition (GC) categories and IC were estimated through a MLR analysis, controlling for age at the time of MMSE assessment, sex, education, smoking status, frequencies of mild, moderate and vigorous physical activities and number of comorbidities. GC was categorized according to MMSE total score thresholds defined previously as *normal*, *mild impairment* and *moderate to severe impairment*. Normal cognition was set as the reference category.

The overall model showed satisfactory fitting (χ^2^(18) = 99.28, *p* < 0.001), with significant effects for IC (χ^2^(2) = 15.23, *p* < 0.001), age (χ^2^(2) = 18.47, *p* < 0.001) and education (χ^2^(2) = 8.01, *p* = 0.018). IC was negatively associated to GC, with significantly increased odds of showing mild impairment over normal cognition in participants with lower IC (aOR = 0.5, *p* < 0.001; 95% aOR CI 0.35, 0.71). However, IC did not predict emerge as associated with increased likelihood of moderate to severe impairment over normal cognition (aOR = 0.217, *p* = 0.62; 95% aOR CI 0.29, 1.32). Conversely, age was positively associated with cognitive impairment, with older ages significantly increasing the likelihood of mild (aOR = 1.07, *p* < 0.001; 95% aOR CI 1.04, 1.12) and moderate to severe cognitive impairment (aOR = 1.1, *p* = 0.018; 95% aOR CI 1.02, 1.19), compared to normal cognition, respectively. Additionally, a lower level of education significantly predicted the risk of mild cognitive impairment over normal cognition (aOR = 0.7, *p* = 0.023; 95% aOR CI 0.52, 0.95), but was not associated with the likelihood of moderate to severe impairment (aOR = 0.54, *p* = 0.057; 95% aOR CI 0.28, 1.02). Although smoking status did not show a significant enough effect at *α* = 0.05 for predicting GC (χ^2^(2) = 5.81, *p* = 0.055), it should be noted that past or current smoker participants had a significantly increased odds of mild cognitive impairment over normal cognition (aOR = 3.49, *p* = 0.026; 95% aOR CI 1.16, 10.47). Sex, engagement in physical activity and the number of comorbidities at the time of IC measurement did not predict later GC. For full details, see Table 4.

**Table 4:** Associations of IC, sociodemographic, lifestyle and comorbidities covariates on global cognition categories (normal cognition set as reference category)

*Summary statistics*	*Model fitting*^*a*^: c^2^(18) = 99.28, p < 0.001				*Goodness of fit:* Pearson *p* = 0.97, Deviance *p* = 1.0				*Pseudo R*^*2*^*:* 0.21^b^	
	Main effects^a^		Mild impairment				Moderate to severe impairment			
	c^2^ (2)	*p*	B (SE)	*p*	aOR	95% CI	B (SE)	*p*	aOR	95% CI
Intrinsic Capacity	15.23	** < 0.001**	−0.699 (0.184)	** < 0.001**	0.5	[0.35, 0.71]	−0.477 (0.386)	0.217	0.62	[0.29, 1.32]
Age	18.47	** < 0.001**	0.071 (0.019)	** < 0.001**	1.07	[1.04, 1.12]	0.095 (0.04)	**0.018**	1.1	[1.02, 1.19]
Sex	0.66	0.721	0.004 (0.261)	0.988	1	[0.6, 1.68]	0.456 (0.574)	0.427	1.58	[0.51, 4.86]
Education	8.01	**0.018**	−0.354 (0.156)	**0.023**	0.7	[0.52, 0.95]	−0.625 (0.328)	0.057	0.54	[0.28, 1.02]
Smoking status	5.81	0.055	1.25 (0.56)	**0.026**	3.49	[1.16, 10.47]	−15.248 (0)	1	0	[0, 0]
Mild physical activity	1.2	0.549	0.012 (0.246)	0.961	1.01	[0.62, 1.64]	−0.536 (0.481)	0.265	0.59	[0.23, 1.5]
Moderate physical activity	0.06	0.969	0.021 (0.19)	0.911	1.02	[0.7, 1.48]	0.096 (0.402)	0.812	1.1	[0.5, 2.42]
Vigorous physical activity	0.8	0.672	−0.136 (0.178)	0.445	0.87	[0.62, 1.24]	−0.203 (0.41)	0.621	0.82	[0.37, 1.82]
Number of comorbidities	4.1	0.129	0.22 (0.113)	0.052	1.25	[1, 1.55]	0.205 (0.241)	0.394	1.23	[0.77, 1.97]

*aOR* adjusted odds ratio

^a^Likelihood ratio tests

^b^Nagelkerke value

To assess whether the lack of associations between IC and moderate/severe cognitive impairment was not due to limited statistical power given the small number of individuals within that particular category (< 3% of the sample) an additional logistic regression was conducted on GC categories collapsing mild and moderate/severe as “impaired GC” and using normal cognition as reference. As shown in Table 5, the strong association of IC and MCI previously found was maintained when collapsing categories with similar significance of the main effect and odds ratio. Interestingly, only after collapsing GC impairment categories, a significant association with number of comorbidities emerged (aOR = 1.24, *p* = 0.042; 95% aOR CI 1.01, 1.53) and the association with smoking status failed to achieve significance.

**Table 5:** Associations of IC, sociodemographic, lifestyle, and comorbidities covariates on global cognition (categories mild, moderate and severe collapsed to Impaired) (normal cognition set as reference category)

*Summary statistics*	Main effects^a^		*Model fitting*^*a*^*: *c^2^*(9)* = *94.69, p* < *0.001 Pseudo R*^*2*^*: 0.21*^*b*^			
			Impaired GC			
	c^2^(1)	*p*	B (SE)	*p*	aOR	95% CI
Intrinsic capacity	14.93	** < 0.001**	−0.665 (0.173)	** < 0.001**	0.51	[0.37, 0.72]
Age	18.12	** < 0.001**	0.075 (0.018)	** < 0.001**	1.08	[1.04, 1.12]
Sex	0.07	0.788	0.066 (0.247)	0.788	1.07	[0.66, 1.73]
Education	7.36	**0.007**	−0.395 (0.146)	**0.007**	0.67	[0.51, 0.9]
Smoking status	3.09	0.079	1.032 (0.56)	0.065	2.81	[0.94, 8.41]
Mild physical activity	0.09	0.763	−0.069 (0.229)	0.762	0.93	[0.6, 1.46]
Moderate physical activity	0.03	0.873	0.029 (0.18)	0.873	1.03	[0.72, 1.46]
Vigorous physical activity	0.75	0.385	−0.144 (0.167)	0.39	0.87	[0.62, 1.2]
Number of comorbidities	4.09	**0.043**	0.217 (0.107)	**0.042**	1.24	[1.01, 1.53]

*aOR* adjusted odds ratio

^a^Likelihood ratio tests

^b^Nagelkerke value

To further explore unadjusted effects of IC and each covariate on mild and moderate/severe cognitive impairment categories, a set of bivariate regressions were conducted using normal cognition as reference (details in Table 6). The bivariate analysis revealed a significant negative association between IC and both mild (aOR = 0.35, *p* < 0.001; 95% aOR CI 0.26, 0.47) and moderate/severe impairment (aOR = 0.35, *p* = 0.001; 95% aOR CI 0.19, 0.65), suggesting that the lack of significance shown in the fully adjusted model between IC and moderate/severe impairment was due to the interaction of IC and other covariates sharing the explained variance. The bivariate regressions also revealed higher odds of progressing from normal cognition to MCI associated with reduced moderate (aOR = 0.66, *p* = 0.005; 95% aOR CI 0.49, 0.88) or vigorous (aOR = 0.62, *p* = 0.003; 95% aOR CI 0.45, 0.85) physical activity and increased number of comorbidities (aOR = 1.42, *p* = 0.001; 95% aOR CI 1.16, 1.74).

**Table 6:** Bivariate associations between IC, covariates and global cognition categories (normal cognition set as reference category)

	*Pseudo*	Main effects^b^		Mild impairment				Moderate to severe impairment			
	*R*^*2 a*^	c^2^(2)	*p*	B (SE)	*p*	aOR	95% CI	B (SE)	*p*	aOR	95% CI
Intrinsic capacity	0.12	54.9	** < 0.001**	−1.052 (0.155)	** < 0.001**	0.35	[0.26,0.47]	−1.052 (0.319)	**0.001**	0.35	[0.19,0.65]
Age	0.11	49.9	** < 0.001**	0.107 (0.017)	** < 0.001**	1.11	[1.08,1.15]	0.124 (0.037)	**0.001**	1.13	[1.05,1.22]
Sex	0.004	0.172	0.4424	0.194 (0.224)	0.386	1.22	[0.78,1.89]	0.519 (0.514)	0.312	1.68	[0.61,4.6]
Education	0.05	23.43	** < 0.001**	−0.554 (0.134)	** < 0.001**	0.58	[0.44,0.75]	−0.802 (0.293)	**0.006**	0.45	[0.25,0.8]
Smoking status	0.01	5.92	0.052	1.253 (0.513)	**0.015**	3.5	[1.28,9.57]	−15.116 (0)	1	0	[0,0]
Mild physical activity	0.005	2.07	0.355	−0.201 (0.198)	0.31	0.82	[0.56,1.21]	−0.449 (0.375)	0.231	0.64	[0.31,1.33]
Moderate physical activity	0.02	9.17	**0.01**	−0.419 (0.151)	**0.005**	0.66	[0.49,0.88]	−0.513 (0.317)	0.105	0.6	[0.32,1.11]
Vigorous physical activity	0.03	12.66	**0.002**	−0.478 (0.16)	**0.003**	0.62	[0.45,0.85]	−0.631 (0.385)	0.102	0.53	[0.25,1.13]
Number of comorbidities	0.03	12.34	**0.002**	0.35 (0.103)	**0.001**	1.42	[1.16,1.74]	0.314 (0.224)	0.162	1.37	[0.88,2.12]

*aOR* adjusted odds ratio

^a^Nagelkerke value

^b^Likelihood ratio tests

Given the significant effects of number of comorbidities on MCI in the bivariate regression (Table 6) and in the fully adjusted model for collapsed cognitive impairment categories (Table 5), we explored whether the lack of associations between GC categories and number of comorbidities found in the first MLR assessment (Table 4) was a true negative or due to a multicollinearity effect. After revealing a strong correlation between IC and the number of comorbidities (Spearman’s correlation rs = −0.198, *p* < 0.001, *n* = 731), a new logistic regression analysis was performed on GC categories excluding number of comorbidities from the fully adjusted model (see details in Table 7). Findings confirmed our primary results, achieving satisfactory model fitting (χ^2^ (16) = 95.2, *p* < 0.001) and showing almost identical significant effects for IC (χ^2^ (2) = 18.69, *p* < 0.001), age (χ^2^ (2) = 18.21, *p* < 0.001), and education (χ^2^ (2) = 7.87, *p* = 0.02), compared to the model including number of comorbidities. Moreover, the significant negative association between IC and MCI (aOR = 0.47, *p* < 0.001; 95% aOR CI 0.33, 0.67), but not between IC and moderate to severe cognitive impairment (aOR = 0.59, *p* = 0.161; 95% aOR CI 0.28, 1.24) was confirmed, with the lack of significant associations with sex and physical activity. Altogether, these results confirm a true association of low IC in cognitively normal older adults and later development of mild cognitive impairment, but not moderate or severe decline.

**Table 7:** Associations of IC, sociodemographic and lifestyle covariates on global cognition categories (number of comorbidities excluded from model, normal cognition set as reference category)

*Summary statistics*	*Model fitting*^*a*^: c^2^(16) = 95.2, p < 0.001				*Goodness of fit:* Pearson *p* = 0.99, Deviance p = 1.0				*Pseudo R*^*2*^*:* 0.196^b^	
	Main effects^a^		Mild impairment				Moderate to severe impairment			
	c^2^(2)	p	B (SE)	*p*	aOR	95% CI	B (SE)	*p*	aOR	95% CI
Intrinsic capacity	18.69	** < 0.001**	−0.762 (0.181)	** < 0.001**	0.47	[0.33, 0.67]	−0.535 (0.382)	0.161	0.59	[0.28, 1.24]
Age	18.21	** < 0.001**	0.071 (0.019)	** < 0.001**	1.07	[1.03, 1.11]	0.094 (0.04)	**0.018**	1.1	[1.02, 1.19]
Sex	0.62	0.732	−0.061 (0.258)	0.812	0.94	[0.57, 1.56]	0.405 (0.571)	0.477	1.5	[0.49, 4.59]
Education	7.87	**0.02**	−0.349 (0.155)	**0.024**	0.71	[0.52, 0.96]	−0.616 (0.326)	0.059	0.54	[0.29, 1.02]
Smoking status	5.47	0.065	1.188 (0.561)	**0.034**	3.28	[1.09, 9.85]	−15.313 (0)	1	0	[0, 0]
Mild physical activity	1.16	0.561	0.047 (0.245)	0.849	1.05	[0.65, 1.69]	−0.507 (0.479)	0.29	0.6	[0.24, 1.54]
Moderate physical activity	0.04	0.981	0.001 (0.19)	0.997	1	[0.69, 1.45]	0.078 (0.401)	0.845	1.08	[0.49, 2.37]
Vigorous physical activity	0.93	0.628	−0.149 (0.178)	0.403	0.86	[0.61, 1.22]	−0.212 (0.41)	0.606	0.81	[0.36, 1.81]

*aOR* adjusted odds ratio

^a^Likelihood ratio tests

^b^Nagelkerke value

A further analysis confirmed significant differences of IC between GC categories (Kruskal–Wallis test: H(2) = 46.73, *p* < 0.001). Participants with mild and moderate to severe cognitive impairment showed significantly lower IC compared to those who maintained normal cognitive function (*normal vs. mild:* Q = 152.94, *p* < 0.001; *normal vs. moderate to severe*: Q = 124.59, *p* = 0.048; Dunn’s test for multiple comparisons). However, no differences were found between mild and moderate to severe categories (Q = −28.35, *p* = 1.00, full details in Table 8).

**Table 8:** Multiple comparison test of IC between global cognition categories

*Kruskal–Wallis test:* H(2) = 46.73, p < 0.001		Dunn’s test for multiple comparisons^a^	
GC category	IC Mean ± SEM	vs. Mild	vs. Moderate to severe
Normal cognition	−0.013 ± 0.642	Q = 152.94, ***p* < 0.001**	Q = 124.59, ***p* = 0.048**
Mild impairment	−0.567 ± 0.764		Q = −28.35, *p* = 1.00
Moderate to severe impairment	−0.567 ± 0.951		

Bold numbers denote significant values with *p* < 0.05

^a^*p* values adjusted by the Bonferroni correction

## Discussion

This study investigated the associations between intrinsic capacity and global cognitive function later in life. We derived two versions of the IC score and tested the sensitivity of results regarding the association between these scores and later global cognition measured using the MMSE. Compared to the z-score derived IC algorithm, the calculation using the IRT method showed better model fitting criteria and thus was chosen to test associations with later global cognition, demographic and lifestyle covariates. Potential collinearities caused by the cognitive domain of IC and the cognitive evaluation performed by MMSE tests were discarded in both algorithms.

Significant associations of IC were found with age, sex and education, such that being a younger male participant with a higher educational level predicted was associated with better IC. Conversely, female sex, older age and lower education were associated with decreased IC, as in line with previous evidence [6, 35].

IC significantly predicted an increased risk of developing mild cognitive impairment 4 to 5 years later. Moreover, participants who maintained normal cognition displayed significantly higher IC scores compared to those who had both mild and moderate to severe cognitive impairment.

After additional regressions to test whether the lack of associations between IC and moderate/severe cognitive impairment was not due to limited statistical power given the small number of individuals within that particular category, we confirmed a true association of low IC in cognitively normal older adults and later development of mild cognitive impairment, but not moderate or severe decline. This specific relation could be explained by the exclusion of individuals diagnosed with MCI or dementia at the time of IC calculation in the selected sample, increasing the probability that most participants would have progressed towards a mild impairment within the following 4 to 5 years, unless they had suffered from an acute neurological condition over this period (i.e. brain stroke or prodromal Alzheimer’s disease). Nonetheless, the inclusion of cognitively normal individuals only was intended as a main objective of our study to test the relationship of previous IC on later cognition without inflating the cognitive domain. Future analysis to compare the predictive value of IC in the trajectory from normal cognition to dementia should be a key step in a follow-up study.

In agreement with previous evidence, an increased likelihood of cognitive decline was also associated with older ages [36] and lower level of education [37, 38], but was not predicted by sex [36], engagement in physical activity [39] or the previous number of comorbidities. Interestingly, when collapsing impaired GC categories, the significant effect of smoking status related to mild impairment shown in the fully adjusted model disappears, suggesting that smoking is particularly associated with that particular cognitive category. Moreover, as the association to mild cognitive impairment is also shown in the bivariate regression, it can be suggested that smoking increased the odds of cognitive decline regardless previous level of the individual’s IC.

Although the present study is not intended to be a prospective or longitudinal analysis, the assessment of the association between IC in a sample of cognitively normal older adults and their cognitive progression measured 4 to 5 years later allows to suggest a potential predictive value of IC on future cognitive decline. However, in a future study we expect to compare GC trajectories associated to IC once ELSA HCAP wave 2 data become available.

Nonetheless, evidence showing a higher predictive validity of IC than number of comorbidities on adverse health outcomes in older adults, such as all-cause mortality and higher risk of fall [40], suggest that our findings related to cognitive decline might be consistent with this trend, particularly given the similar approach to obtain IC scores from clinically validated cut-off thresholds. Moreover, a recent prospective study in the UK Biobank cohort examined the association of baseline IC and the risk of developing future dementia [41] using a similar dichotomic scoring approach based on Beard, 2019 criteria [6], showing that individuals with lower IC have a significantly higher risk of new onset all-cause dementia, particularly vascular and Alzheimer’s disease. Thus, we believed that existing evidence supports the conceptual validity of our proposal and consistency of our findings.

### Limitations and perspectives

The concept of IC highlights the connectedness of the domains to each other and to cognition, and the importance of promoting good health in all aspects of life, not just in cognition, to predict healthy ageing. However, variable selection may be dictated by the availability and completeness of data. In this study, 12 variables were used to generate the IC score, similar to both Campbell [8] and Beard [6], who used 12 and 14 variables respectively. Both studies were performed on the same cohort selected for our assessment but used data from different waves (wave 2 and 4, respectively). Therefore, a main limitation of our study to be compared to previous findings in the ELSA cohort is that IC domains were computed using indicators available at each wave which, unfortunately, were not consistent at each time of assessment. These differences on available data at different assessment times on the same cohort make it impossible to compare between derived IC scores for longitudinal analysis and stress the importance to keep consistency when evaluating participants across time points, and also the need to determine a consensual definition of indicators to define IC domains.

An additional limitation refers to the use of self-assessment scales as measures of IC components in older adults. Particularly for the calculation of the Cognitive Domain, the use of objective sleep measurement should be preferred over subjective self-reports, given the risk of self-recall bias [42]. Moreover, comparative studies between self-reported sleep assessments such as the Pittsburgh Sleep Quality Index (PSQI), and objective sleep measurements in adults over 55 years old have not only failed to show significant correlations, but also revealed that sex, education, age and cognitive status do not influence the lack of associations [42, 43]. The inclusion of wearable devices for objective sleep tracking in clinical research have dramatically increased, leading to substantial improvements in their quality, reliability and validation against gold-standard polysomnography (PSG), allowing to perform multiple consecutive assessment with minimum discomfort assessment in large cohorts of individuals. Future assessments of IC should make use of these valuable tools for objective assessments, particularly now that actigraphy-derived sleep parameters are becoming available in large longitudinal study datasets such as ELSA wave 10 and UK Biobank.

To date, this is the first study reporting the association between IC and later probability of developing cognitive decline in a sample of older adults. IC has the potential to be a valuable predictive construct, as it is more personalised than age [44], particularly in older participants where abilities differ hugely within the population. In this line, a recent randomized trial in cognitively normal community-dwelling older adults with impaired IC in ≥ 3 subdomains at baseline, showed that a 12-month multidomain intervention based on individual IC deficits prevented cognitive decline and physical frailty [45]. Therefore, the clinical relevance of an adequate measurement of IC transcends its value as a mere predictor of age-related functional to become a novel screening tool to design personalized strategies for healthy ageing.

Adding IC to existing general health checks could therefore provide a reliable indicator of future cognitive function and help to monitor interventions to improve healthy ageing. Aspects to take into consideration would be the minimum number of variables required for accurate IC calculations, as well as which ones are the fastest and easiest to measure; in other words, figuring out the most practicable and efficient way of calculating IC. Furthermore, future work may consider the different contribution of each variable and domain to the IC calculation. Alongside this, there is scope to develop interventions to improve and maintain a high IC, so that these findings can be applied to mitigate cognitive decline.

## Data Availability

The data used in this study is freely available and can be accessed through the UK Data Service at 10.5255/UKDA-SN-5050–31 for the English Longitudinal Study of Ageing: Waves 0–10, 1998–2023, and at 10.5255/UKDA-SN-8502–4 for the English Longitudinal Study of Ageing: Harmonised Cognitive Assessment Protocol, 2018–2023.
